# Successful transcatheter edge-to-edge repair for atrial functional mitral regurgitation after surgical annuloplasty ring dehiscence: a case report

**DOI:** 10.1093/ehjcr/ytae396

**Published:** 2024-08-06

**Authors:** Ryota Kosaki, Yasuhide Mochizuki, Eiji Toyosaki, Hiroto Fukuoka, Toshiro Shinke

**Affiliations:** Division of Cardiology, Department of Medicine, Showa University School of Medicine, 1-5-8 Hatanodai, Shinagawa-ku, Tokyo 142-8555, Japan; Division of Cardiology, Department of Medicine, Showa University School of Medicine, 1-5-8 Hatanodai, Shinagawa-ku, Tokyo 142-8555, Japan; Division of Cardiology, Department of Medicine, Showa University School of Medicine, 1-5-8 Hatanodai, Shinagawa-ku, Tokyo 142-8555, Japan; Division of Cardiology, Department of Medicine, Showa University School of Medicine, 1-5-8 Hatanodai, Shinagawa-ku, Tokyo 142-8555, Japan; Division of Cardiology, Department of Medicine, Showa University School of Medicine, 1-5-8 Hatanodai, Shinagawa-ku, Tokyo 142-8555, Japan

**Keywords:** Atrial functional mitral regurgitation, Edge-to-edge mitral repair, Annuloplasty ring dehiscence, Transoesophageal echocardiography, Case report

## Abstract

**Background:**

Annuloplasty ring dehiscence (ARD) after surgical mitral valve repair is a rare complication, which causes recurrent mitral regurgitation (MR) and is associated with adverse outcomes in patients with a prohibitive risk of repeat surgery. However, a patient developed severe MR, when challenging transcatheter edge-to-edge repair (TEER) after surgical ring dehiscence, it should be considering the relative efficacy and safety.

**Case summary:**

An 89-year-old man underwent mitral valve repair with an annuloplasty ring for moderate atrial functional MR (AFMR). Post-operative transthoracic echocardiography on Day 7 suggested a dislodged mitral annuloplasty ring and recurrent moderate AFMR. However, the MR developed severely, which led to two hospitalizations for congestive heart failure in the past year. Transoesophageal echocardiography (TOE) was performed carefully to ensure that the TEER clip did not interfere with the dislodged annuloplasty ring. Consequently, only the therapeutic target on the medial side of the A2–P2 region was approached posteriorly behind the peri-ring space, without gripper interference.

**Discussion:**

Transcatheter edge-to-edge repair using the G4-MitraClip® system is feasible and safe in patients with recurrent severe AFMR after surgical mitral valve repair concomitant with ARD. Meticulous simulation with pre-operative TOE is one of the crucial steps for successful outcomes.

**Learning points**

- The annuloplasty ring dehiscence (ARD) after surgical mitral valve repair is a rare complication that can cause recurrent mitral regurgitation (MR).
- Transcatheter edge-to-edge repair for recurrent MR due to ARD is one of the potential therapeutic options for high-surgical-risk patients, but it is crucial to thoroughly simulate the approach method pre-operatively.

## Introduction

Recurrent mitral regurgitation (MR) after surgical mitral valve repair worsens prognosis.^1^ Annuloplasty ring dehiscence (ARD) after surgical mitral valve repair is a rare complication, which can cause MR recurrence.^2^ Here, we discuss a case of atrial functional MR (AFMR) in which the coaptation of the ‘flat’ mitral valve was adjacent to the detached ring in systole. Transcatheter edge-to-edge repair (TEER) is one of the treatment strategies for recurrent MR associated with ARD. A pre-operative anatomical evaluation of the potential interference between the clip arms or grippers and the ring in TEER enabled the implementation of the procedure.

## Summary figure

**Table:** 

Date	Events
May 2015	Diagnosis of heart failure with chronic atrial fibrillation and moderate atrial functional mitral regurgitation and severe tricuspid regurgitation.
11 November 2015	Cardiac surgery: mitral annular repair (28 mm Cosgrove annuloplasty ring) with A3/P3 plication suture and tricuspid annuloplasty (32 mm MC annuloplasty ring). Transthoracic echocardiography on the seventh post-operative day showed the dislodged mitral annuloplasty ring.
24 February 2016	Transvenous implantation of a permanent pacemaker for symptomatic bradycardia with atrial fibrillation.
May and September, 2022	Two-time hospitalizations for congestive heart failure due to severe atrial functional mitral regurgitation.
20 October 2022	Transcatheter edge-to-edge mitral valve repair using one NT clip that is MitraClip G4 system.
July 2023	Nine months later, transthoracic echocardiography showed that the implanted NT clip was stable, residual mitral regurgitation was mild, and his NYHA functional class was II.

## Case presentation

**Figure 1:**
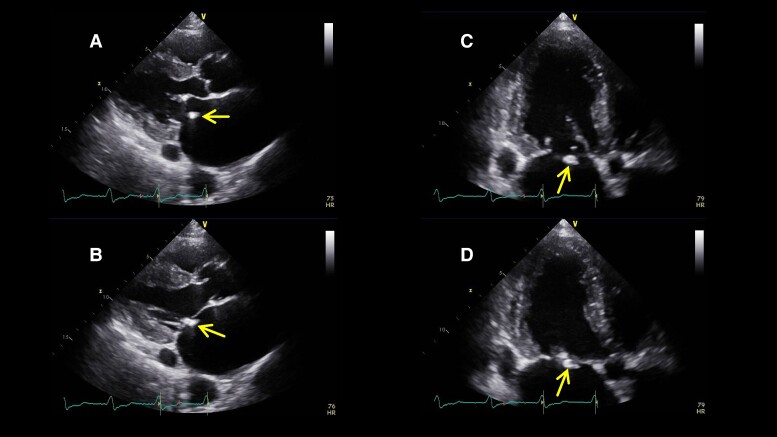
Parasternal long axis view in end-diastole (*A*) and end-systole (*B*) and apical long axis view in end-diastole (*C*) and end-systole (*D*) with the detached annuloplasty ring (arrow) on transthoracic echocardiography.

**Figure 2:**
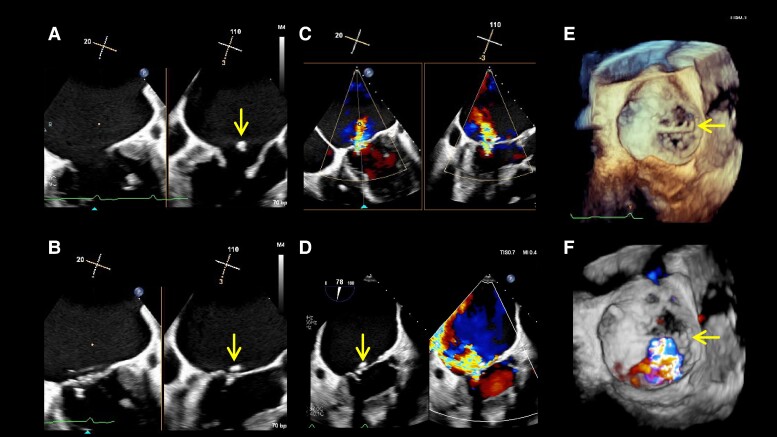
Multiplane view presenting the commissural view (left: 20°) and left ventricular out tract view (right: 110°) in transoesophageal echocardiography (TOE) (*A*: end-diastole, *B*: end-systole, and arrows: detached annuloplasty ring). Detached annuloplasty ring and atrial functional mitral regurgitation (*C* and *D*) and its three-dimensional TOE image (*E* and *F*).

**Figure 3:**
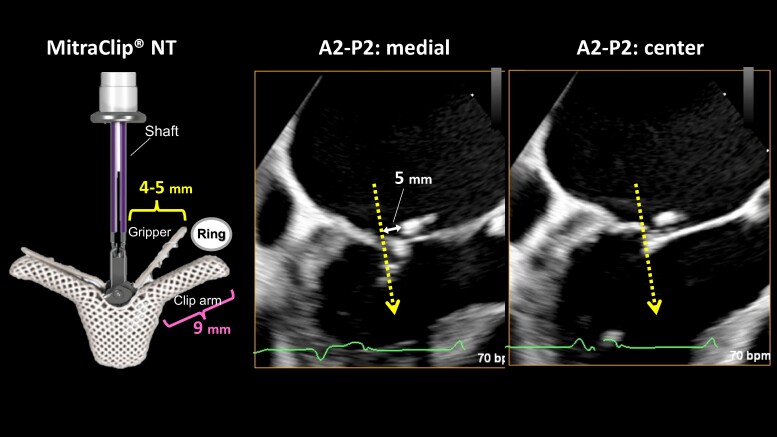
Positional relationship of the clip presumed to be the detached annuloplasty ring and the anticipated direction of clip insertion (dashed arrow); on the A2–P2 medial side, there is a possible 5 mm space between the dislodged ring and the shaft.

**Figure 4:**
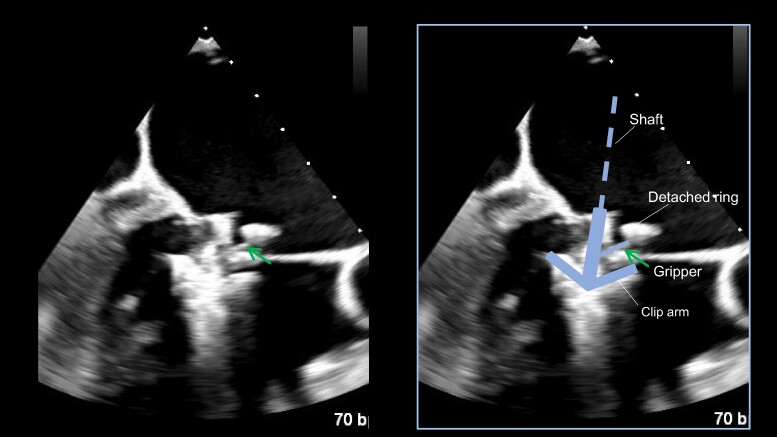
Clip inserted into the left ventricle in the intraoperative transoesophageal echocardiography (TOE) and success of the anterior gripper (arrow) down without interfering with the ring (*A*). Fusion of the real TOE image and schematic (*B*).

**Figure 5:**
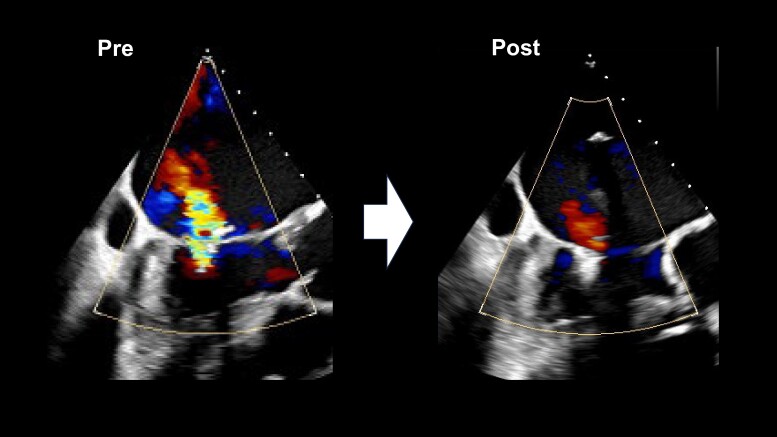
Intraoperative transoesophageal echocardiography (TOE) images before (left) and after transcatheter edge-to-edge repair (right) with improved mitral regurgitation.

An 89-year-old man underwent mitral valve annuloplasty (MAP) with a 28 mm Cosgrove annuloplasty ring for moderate AFMR associated with long-standing atrial fibrillation and enlarged atria and tricuspid valve annuloplasty (TAP) for severe tricuspid regurgitation (TR) with a 32 mm MC annuloplasty ring in November 2015. Post-operative transthoracic echocardiography (TTE) on Day 7 indicated a dislodged mitral annuloplasty ring and moderate AFMR recurrence, with controlled TR. Thus, a watchful waiting strategy was adopted, and 7 years passed without recurrence of heart failure (HF). However, in 2022, the patient was hospitalized twice for HF with the New York Heart Association (NYHA) Class III symptoms under the following medications for HF: azosemide (30 mg), torasemide (4 mg), tolvaptan (15 mg), bisoprolol (0.625 mg), valsartan (40 mg), and empagliflozin (10 mg). On admission for congestive HF, physical examination suggested an oxygen saturation of 94% on room air and bilateral pretibial oedema. Auscultation suggested coarse crackles in the lung fields and a pan-systolic murmur of Levine grade II at the apex. His blood pressure was 115/57 mmHg, pulse rate was 84 b.p.m., and a 12-lead electrocardiogram indicated right ventricular pacing of 84 b.p.m. The chest X-ray suggested cardiac dilation (cardiothoracic ratio = 65%), pulmonary congestion in the lung fields, and bilateral pleural effusion. Blood exam indicated an elevated N-terminal pro-B-type natriuretic peptide level of 2476 pg/mL. On TTE, the left ventricular (LV) end-diastolic diameter had increased to 54 mm, with a preserved LV ejection fraction of 62% (end-diastolic/end-systolic volume = 143/54 mL). A detached ring was observed in the left atrium at the mitral annulus level (*Figure 1*). The left atrial volume index suggested substantial enlargement at 139 mL/m^2^. Transthoracic echocardiography demonstrated worsened AFMR with an effective regurgitant orifice area of 0.35 cm^2^ and a regurgitant volume of 55.4 mL (*Movie Clip A*). The TR was controlled to be mild. Transoesophageal echocardiography (TOE) indicated a ‘flat valve’ with a shallow coaptation of the mitral valve (MV) with partial pseudo prolapse. Moreover, the MV coaptation was located immediately below the dislodged prosthetic annulus ring; the MR jet collided with the displaced posterior ring (*Figure 2*, *Movie Clip B*). Three-dimensional TOE analysis indicated an MV area of 3.9 cm^2^. He was deemed to be at a relatively high surgical risk, with STS scores of 7.3% and 12.9% for valve repair and replacement, respectively. Therefore, TEER was selected as the final treatment strategy for the cardiology team discussions. The following issues were deliberated because the MV coaptation almost touched the detached ring during systole: (1) route of entry of the clip delivery system (CDS), whether it should enter from the anterior space inside the ring or posterior peri-ring space; (2) a distance of ∼5 mm between the shaft and ring required to lower the gripper down without interfering with the ring; and (3) whether the clip arm could be closed without touching the ring. From the TOE analysis, we decided to approach the flat MV vertically by pushing the CDS through the peri-ring space with the shaft away from the aorta ‘reverse aorta hugger’. Therefore, the septal puncture was planned to be performed as posteriorly as possible. Moreover, a distance of ∼5 mm between the shaft and ring could be ensured on the medial side of A2–P2 to lower the gripper down (*Figure 3*). In addition, the length of the P2 segment, which was the target for TEER, was 10.7 mm, and the mean trans-mitral gradient was elevated at 4.0 mmHg. For these reasons, it was determined that the only device option capable for TEER in this case was NT clip. Successful MV grasping was achieved by the planned entry of the NT (*Figure 4*), as indicated by the intra-procedural TOE. Subsequent positioning optimization by steering down in the antero-inferior direction closed the clip arm, resulting in a substantial reduction in MR (*Figure 5* and *Movie Clip C*). The patient’s post-operative course was uneventful, and the NYHA functional class at 9 months was II. Transthoracic echocardiography suggested a stable clip and mild residual MR. To date, the patient has not been admitted for HF-related hospitalization during the subsequent 1.5 years.

## Discussion

This case involved a patient who underwent MAP and TAP for severe TR combined with moderate AFMR 7 years ago. Annuloplasty ring dehiscence was confirmed 1 week after surgery; nonetheless, the patient was managed conservatively for several years. After 7 years, TEER was performed successfully for congestive HF caused by AFMR. The uniqueness of this report lies in AFMR recurrence, with the detached ring positioned adjacent to the MV coaptation and meticulous planning performed before the procedure.

A report from a high-volume single institution between 1996 and 2016 of 3478 patients who underwent valvuloplasty and annuloplasty for degenerative MR showed that 57 (1.6%) patients experienced ARD, of which 44% occurred within 1 month and largely in the posterior regions. The 30-day mortality after reoperation for MV was 2%, with 1- and 5-year survival rates of 89% and 74%, respectively. During reoperation, MV replacement was performed in 38 cases (67%), whereas MV re-repair was performed in 19 cases (33%).^2^ Transcatheter edge-to-edge repair has been widely recognized as an effective, safe, and proven catheter-based treatment for MR in patients at high surgical risk; it has established efficacy and long-term outcomes.^3–5^ However, only few reports have elucidated TEER for recurrent MR with ARD.^6–9^ According to a report from a single centre, of 795 patients with TEER, six patients with ARD underwent treatment using the MitraClip system (Abbott, Santa Clara, CA, USA), and all six achieved technical success.^7^ Leurent *et al.* demonstrated that technical success rate of the TEER was 100% in 23 patients with recurrent MR after surgical mitral annuloplasty from the multicentre ‘Clip-in-Ring’ registry. At discharge, residual MR grade was ≤2+ in 87% and median trans-mitral gradient was 4 [3–5] mmHg.^10^ Therefore, TEER can be recognized as a technically feasible option for treating MR recurrence caused by ARD. Patients with recurrent MR from ARD have poor prognosis; however, TEER can be performed with ingenuity to improve the symptoms and avoid readmission for HF. In addition, transcatheter mitral valve implantation (TMVI) may become one of the treatment options for recurrent MR after mitral valve surgery.^11^ In fact, a case of TMVI performed for recurrent MR with ARD was reported.^12^ Our report highlights a relatively rare case of TEER in which the CDS was approached from the posterior of the peri-ring and perpendicular to the MV ‘reverse aorta hugger approach’. This is because posterior of the detached ring was located anteriorly distant from the norm. Depending on the degree of ring dislodgement, it may be preferable to deliver the CDS with an ‘aorta hugger’ through the ring.^7^ Therefore, pre-operative anatomical evaluation is necessary.

Furthermore, AFMR with a giant left atrium was the primary aetiology of MR in this case. The gripper and the opening and closing of the clip arm could interfere with the ring because the MV coaptation was adjacent to the ring. Therefore, it was necessary to simulate the insertion position and gripper down within a few millimetres. The strength of this paper lies in the detailed pre-operative meticulous planning conducted under TOE guidance.

## Conclusion

Recurrent AFMR with ARD is rare; however, TEER is a feasible procedure with a well-developed pre-procedural strategy using TOE.

## Lead author biography


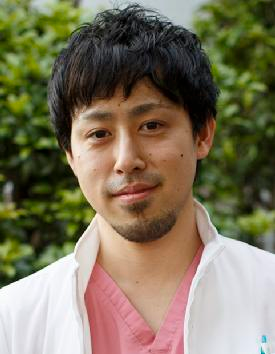


Dr Ryota Kosaki graduated from Aichi Medical University in 2012 and completed doctoral programme at Showa Graduate School of Medicine. He works at Showa University Hospital and specializes in catheter intervention for ischaemic heart disease and structural heart disease.

## Supplementary Material

ytae396_Supplementary_Data

## Data Availability

The data underlying this article will be shared upon reasonable request with the corresponding authors.
